# A visceral mycosis in farmed rainbow trout (*Oncorhynchus mykiss*) caused by *Neopyrenochaeta submersa*

**DOI:** 10.1016/j.mmcr.2023.05.001

**Published:** 2023-05-19

**Authors:** Jiří Řehulka, Alena Kubátová, Vit Hubka

**Affiliations:** a Department of Zoology, Silesian Museum, 746 01, Opava, Czech Republic; b Department of Botany, Faculty of Science, Charles University, 128 00, Prague 2, Czech Republic; c Laboratory of Fungal Genetics and Metabolism, Institute of Microbiology of the Academy of Sciences of the Czech Republic, v.v.i., 142 20, Prague 4, Czech Republic

**Keywords:** Coelomycetous fungi, Histopathology, Molecular diagnosis, *Phoma*, *Pyrenochaeta*

## Abstract

A mycotic infection manifesting as abdominal distension with free serous fluid accumulation in the coelomic cavity is documented in farmed rainbow trout. Histological examination using PAS and silver staining revealed the presence of numerous fungal hyphae in the spleen and gastrointestinal wall. The isolated fungus was sterile and identified by using phylogenetic analysis based on four loci as *Neopyrenochaeta submersa*. This is the first time this fungus has been reported as pathogen.

## 1 Introduction

Protection of fish in intensive culture against infections of bacterial or mycotic origin is among the key preventive measures within the range of the key principles of veterinary health management to minimize the morbidity of the fish. Routine use of antibiotics and their combinations, which all affect the immunity mechanisms, results in an increasing incidence of opportune mycotic infections. In immunodeficient hosts they may lead to a severe manifest illness in the form of generalized infection, induced either through activation of a latent infection from an endogenous infection source, or through transfer of an exogenous pathogen to the host from a source in the external environment. Colonization of the mucous membrane of the digestive tract is a significant endogenous source of the mycotic agent and a risk factor underlying the rise of disseminated mycosis. In salmonid aquaculture there is a trend of increasing incidence of visceral mycotic infections in farmed rainbow trout. The spectrum of primary etiological agents of serious health disorders also expanded in the last years [[1], [2], [3]].

Coelomycetous fungi are relatively rare and poorly studied human and animal pathogens with genera *Phoma*, *Pyrenochaeta* and *Neoscytalidium* being the most commonly reported pathogens [4]. Valenzuela-Lopez et al. [5] proposed a new genus *Neopyrenochaeta* and new family *Neopyrenochaetaceae* encompassing several taxa previously included in *Pyrenochaeta*. Pathogenic *Pyrenochaeta* spp. were mostly accommodated in other genera such as *Neocucurbitaria* (*N. keratinophila*, *N. unguis-hominis*), *Medicopsis* (*M. romeroi*) and *Nigrograna* (*N. mackinnonii*) [4,5]. Thus, current members of the genus *Neopyrenochaeta* are predominantly saprophytes and plant pathogens which are non-pathogenic for human and animals. As far as we know, the only exception is the report of swim bladder infection in pacific salmon caused by *Neopyrenochaeta acicola* (syn. *Pyrenochaeta acicola*) [6]. Other cases of infection in fish due to coelomycetous fungi are mostly restricted to the genera *Phoma* or *Phaeophleospora* [7,8].

This report describes the first case of infection by a recently described species of *Neopyrenochaeta*, *N. submersa*, isolated from a young, farmed rainbow trout, *Oncorhynchus mykiss* Walbaum. The fungal pathogen was identified using morphological and molecular data. To our knowledge, this is the first case of infection due to this fungal species in animals.

## 2 Case presentation

**Fig. 1:**
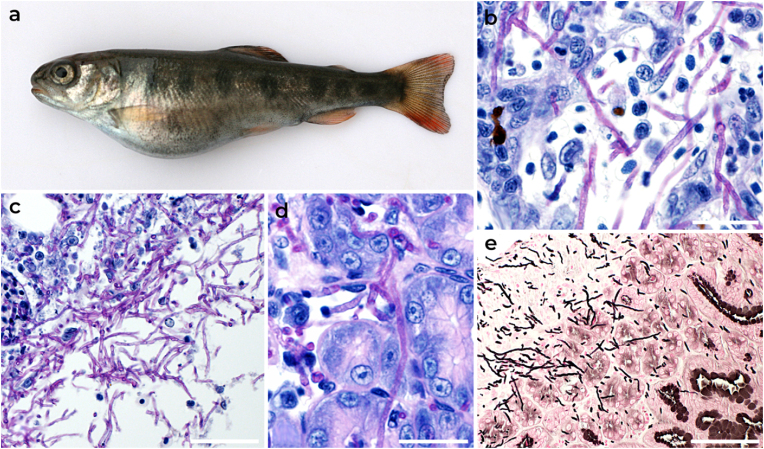
Spontaneous infection in rainbow trout caused by *Neopyrenochaeta submersa*. Abdominal distension in rainbow trout (standard body length 115 mm) due to ascites (a); foci of fungal hyphae in the sinusoids of the spleen (b); high number of fungal hyphae invading the wall of the intestine (c) and high-power view of hyphae (d); fungal hyphae invading gastrointestinal wall (e); Periodic acid–Schiff (b–d), Grocott’s methenamine silver stain (e). Scale bar: b, d = 10 μm, c, e = 20 μm.

The disease occurred in one of the fifteen rainbow trout subjected to post-mortem examination during a routine health check on a commercial fish farm (404 m above sea level in the watershed of the River Odra, Czech Republic). The fish were reared in concrete tanks and kept at a density of 100 kg m^-3^. The water had the following physical and chemical characteristics: temperature 7 °C, dissolved O_2_ 9 mg L^-1^, pH 7.1, total hardness 6.5°N, chemical oxygen demand (COD_Mn_) 2.7 mg L^-1^, nitrites (NO_2_^-^) 0.062 mg L^-1^ and nitrates (NO_3_^-^) 5.9 mg L^-1^. The fish had a standard length of 115 mm and was fed dry pellets containing 40% crude protein and 14% crude fat. Clinical signs included abdominal distension (Fig. 1a), and at necropsy the coelomic cavity contained serous fluid. Microscopic wet mount examination of the spleen and intestine contents revealed abundant fungal hyphae. Histological examination showed foci of PAS positive fungal hyphae in the spleen and numerous fungal hyphae invading the gastrointestinal wall (Fig. 1b–e), no fungal elements were found in gill, liver, kidney, heart, and brain. Histological findings showed the fungus’ tendency to penetrate the surrounding tissue and induce systemic mycosis. A fungal strain was isolated in pure culture from the spleen and intestine on Sabouraud’s agar at 24 °C. Bacteria were not observed in the lesion material and were not cultivated from samples inoculated on Blood agar (Columbia Agar Base; Merck), trypticase soya agar (Oxoid Ltd), Mueller-Hinton agar and Anacker-Ordal agar. Parasitological investigation revealed only minor infestation by *Gyrodactylus bohemicus* (the intensity of infestation on fins was three specimens).

**Fig. 2:**
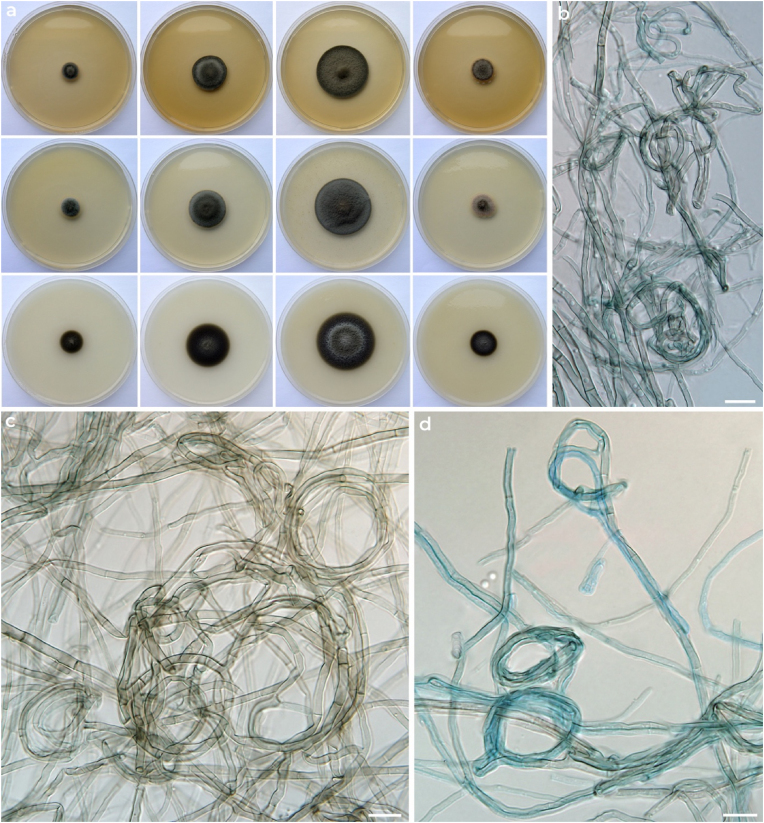
Macromorphology and micromorphology of *Neopyrenochaeta submersa* CCF 5741. Colonies incubated for 14 days on MEA, PDA and OA (rows, top to bottom) at 10, 15, 20 and at 25 °C (columns, left to right) (a). Sterile vegetative hyphae, hyaline to brown, occasionally forming hyphal coils (b–d). Scale bars: b-d = 10 μm. (For interpretation of the references to colour in this figure legend, the reader is referred to the Web version of this article.)

**Fig. 3:**
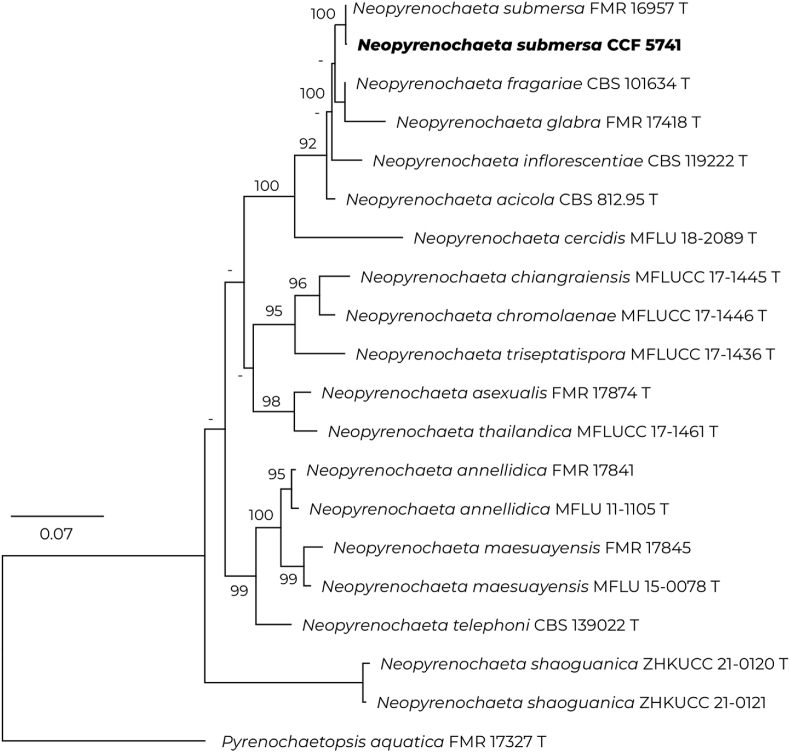
Best-scoring maximum-likelihood tree based on combined data from ITS and LSU rDNA regions, β-tubulin (*tub2*) and RNA polymerase II second largest subunit (*rpb2*) genes, showing the relationships of case isolate of *Neopyrenochaeta submersa* CCF 5741 to other *Neopyrenochaeta* species. The dataset was partitioned, models were selected (ITS: TNe + G4; LSU: K2P + I; *tub2*: TNe + G; *rpb2*: TIM2e + I + G4) and tree constructed using IQ-TREE v. 1.6.12 [10]. Only bootstrap support ≥70% are shown; lower supports are designated by a dash; the ex-type strains are designated by a letter "T".

Subcultures of isolated fungal species were grown on malt extract agar (MEA), potato-dextrose agar (PDA) and oatmeal agar (OA) at 10, 15, 20, 25 and 30 °C (Fig. 2a). Colonies very similar on all media: greyish brown, velvety, centrally raised to umbonate with regular margins. Colony diameters after 14 days were as follows: 5–7 mm (10 °C), 10–13 mm (15 °C), 16–19 mm (20 °C), 7–9 mm (25 °C), no growth at 30 °C. The fungus remained sterile even after prolonged cultivation of 2 months and only dark-pigmented vegetative hyphae were observed (Fig. 2b–d). No sporulation was induced on water agar or V8-agar supplemented with sterile banana leaves or grains. Similarly, the fungus remained sterile when incubating some cultures under near-ultraviolet light (12 h light, 12 h dark) [9]. BLAST similarity search with ITS region of rDNA showed affinity of the fungus to the species of *Neopyrenochaeta*. Additional phylogenetic marker genes used in the taxonomy of *Phoma*-like genera [5] were amplified as described previously [7] and phylogenetic tree was constructed (Fig. 3). The fungus was identified as *Neopyrenochaeta submersa*. The sequences were deposited to the GenBank database under following accession numbers: OK329892 (ITS), OK329942 (LSU), OK329943 (SSU), OK334131 (*tub2*) and OK334130 (*rpb2*). The fungus was deposited into the Culture Collection of Fungi, Charles University, Department of Botany, Prague, Czech Republic as CCF 5741, and Westerdijk Fungal Biodiversity Institute, Utrecht, the Netherlands as CBS 148472.

## 3 Discussion

The coelomycetous fungi involved in mycoses are poorly known due to complexity of their identification and relatively low frequency of infections. However, they are responsible for a large variety of clinical entities from superficial to deep and systemic mycoses [11]. Previously reported fish infections were mostly attributed to coelomycete genus *Phoma* and reviewed by Řehulka et al. [7]. Other coelomycete genera are rarely reported as fish pathogens [8]. Until now, only single study reported genus *Neopyrenochaeta* as animal pathogen, namely, *N. acicola* has been reported in fish [6]. Marchenko [6] ranked *N. acicola* among seven species of fungi that caused swim bladder mycosis in a farmed fingerling pacific salmon, especially pink salmon (*Oncorhynchus gorbuscha*) and chum salmon (*Oncorhynchus keta*). Histologically, fungal hyphae were found in the posterior kidney and in some cases protruding into the lumen of intestine.

The infection of spleen, such as found in our study, is relatively common in fish. The spleen is often a harbor for infection, threatening to spread to other organs, including the gut. We think that the administration of quinoline-based wide-spectrum antibiotics might cause changes in the intestinal microflora, facilitating the propagation of the fungus, which then extensively colonized the intestine and grew into adjacent tissues.

In this study, we extended the spectrum of coelomycetous fungi pathogenic for fish. Although our attempts to find characteristic fruiting bodies in isolated fungus were not successful, phylogenetic analysis clearly identified it as a recently described species of *Neopyrenochaeta*, *N. submersa*, described from plant debris in freshwater in Spain [12]. Problems with induction of sporulation in colelomomycetous fungi are relatively common and make their identification based on phenotype challenging or impossible [11]. In these cases, the use of molecular techniques is the only possibility to achieve reliable identification.

## Funding sources

This work was financially supported by the Ministry of Culture of Czech Republic through institutional financing of long-term conceptual development of the research institution (the Silesian Museum, MK000100595) and internal grant of the Silesian Museum No. IGS 201708/2017. Contribution of VH was supported by 10.13039/501100004240Czech Academy of Sciences Long-term Research Development Project (RVO: 61388971).

## Consent

Authors have obtained written and signed consent to publish the case report from legal guardian. Necessary certificate was obtained by the first author (Act No. 167, registration number 0155/2000— V 3). The research conducted in this work was supported by internal grant “Research on systemic fish diseases IV” (project No. IGS 201708/2017) that were approved by the committee DKRVO, MK000595**.** A copy is available upon request.

## Declaration of competing interest

None.

## References

[1] Hubka V., Réblová M., Řehulka J., Selbmann L., Isola D., de Hoog S.G. (2014). *Bradymyces* gen. nov (Chaetothyriales, Trichomeriaceae), a new ascomycete genus accommodating poorly differentiated melanized fungi. Antonie Leeuwenhoek.

[2] Řehulka J., Kubátová A., Hubka V. (2016). *Cephalotheca sulfurea* (Ascomycota, Sordariomycetes), a new fungal pathogen of the farmed rainbow trout *Oncorhynchus mykiss*. J. Fish. Dis..

[3] Řehulka J., Kolařík M., Hubka V. (2020). Clinical and histopathological changes in rainbow trout *Oncorhynchus mykiss* experimentally infected with fungus *Bradymyces oncorhynchi*. Folia Microbiol..

[4] Chowdhary A., Meis J., Guarro J., De Hoog G., Kathuria S., Arendrup M. (2014). ESCMID and ECMM joint clinical guidelines for the diagnosis and management of systemic phaeohyphomycosis: diseases caused by black fungi. Clin. Microbiol. Infect..

[5] Valenzuela-Lopez N., Cano-Lira J., Guarro J., Sutton D.A., Wiederhold N., Crous P. (2018). Coelomycetous *Dothideomycetes* with emphasis on the families *Cucurbitariaceae* and *Didymellaceae*. Stud. Mycol..

[6] Marchenko A.M. (1988). Griby – vozbuditeli mikozov ryb na rybovodnykh zavodakh Sakhalina. Mikol. Fitopatol..

[7] Řehulka J., Kubátová A., Hubka V. (2020). Swim bladder mycosis in farmed rainbow trout *Oncorhynchus mykiss* caused by *Phoma herbarum* and experimental verification of pathogenicity. Dis. Aquat. Org..

[8] Řehulka J., Kubátová A., Hubka V. (2018). Swim bladder mycosis in pretty tetra (*Hemigrammus pulcher*) caused by *Exophiala pisciphila* and *Phaeophleospora hymenocallidicola*, and experimental verification of pathogenicity. J. Fish. Dis..

[9] Su Y.-Y., Qi Y.-L., Cai L. (2012). Induction of sporulation in plant pathogenic fungi. Mycology.

[10] Nguyen L.-T., Schmidt H.A., von Haeseler A., Minh B.Q. (2015). IQ-TREE: a fast and effective stochastic algorithm for estimating maximum-likelihood phylogenies. Mol. Biol. Evol..

[11] Valenzuela-Lopez N., Sutton D.A., Cano-Lira J.F., Paredes K., Wiederhold N., Guarro J. (2017). Coelomycetous fungi in the clinical setting: morphological convergence and cryptic diversity. J. Clin. Microbiol..

[12] Magaña-Dueñas V., Stchigel A.M., Cano-Lira J.F. (2021). New coelomycetous fungi from freshwater in Spain. J Fungi.

